# Injury Incidence Across the Menstrual Cycle in International Footballers

**DOI:** 10.3389/fspor.2021.616999

**Published:** 2021-03-01

**Authors:** Dan Martin, Kate Timmins, Charlotte Cowie, Jon Alty, Ritan Mehta, Alicia Tang, Ian Varley

**Affiliations:** ^1^School of Sport and Exercise Science, University of Lincoln, Lincoln, United Kingdom; ^2^The Football Association, London, United Kingdom; ^3^Department of Sport and Exercise Science, Nottingham Trent University, Nottingham, United Kingdom

**Keywords:** epidemiology, menstrual cycle, injury, soccer, football, female athlete

## Abstract

**Objectives:** This study aimed to assess how menstrual cycle phase and extended menstrual cycle length influence the incidence of injuries in international footballers.

**Methods:** Over a 4-year period, injuries from England international footballers at training camps or matches were recorded, alongside self-reported information on menstrual cycle characteristics at the point of injury. Injuries in eumenorrheic players were categorized into early follicular, late follicular, or luteal phase. Frequencies were also compared between injuries recorded during the typical cycle and those that occurred after the cycle would be expected to have finished. Injury incidence rates (per 1,000 person days) and injury incidence rate ratios were calculated for each phase for all injuries and injuries stratified by type.

**Results:** One hundred fifty-six injuries from 113 players were eligible for analysis. Injury incidence rates per 1,000 person-days were 31.9 in the follicular, 46.8 in the late follicular, and 35.4 in the luteal phase, resulting in injury incidence rate ratios of 1.47 (Late follicular:Follicular), 1.11 (Luteal:Follicular), and 0.76 (Luteal:Late follicular). Injury incident rate ratios showed that muscle and tendon injury rates were 88% greater in the late follicular phase compared to the follicular phase, with muscle rupture/tear/strain/cramps and tendon injuries/ruptures occurring over twice as often during the late follicular phase compared to other phases 20% of injuries were reported as occurring when athletes were “overdue” menses.

**Conclusion:** Muscle and tendon injuries occurred almost twice as often in the late follicular phase compared to the early follicular or luteal phase. Injury risk may be elevated in typically eumenorrheic women in the days after their next menstruation was expected to start.

## Introduction

With the professionalization of women's football, training, and match demands have significantly increased in recent years (Datson et al., 2014, 2017). The overall injury incidence is similar to male football, although the proportion of severe injuries has been shown to be higher in women's football (Mufty et al., 2015; Roos et al., 2017) which is associated with significant costs (Gebert et al., 2020). Female football players are reported to have 21% more absence due to injury compared to men, primarily due to greater incidence of severe knee and ankle ligament injuries, with anterior cruciate ligament (ACL) injuries occurring 2–8 times more often in female soccer players (Larruskain et al., 2018; Lin et al., 2018).

The menstrual cycle has been theorized as a factor which could modify injury risk in female athletes, as cyclical fluctuations in reproductive hormones such as estrogen and progesterone can influence musculoskeletal tissues such as muscle, tendon, and ligament (Herzberg et al., 2017; Chidi-Ogbolu and Baar, 2019). Several studies have identified a greater risk of ACL injury occurrence in the late follicular/ovulatory phase when estrogen concentrations are highest (e.g., Wojtys et al., 2002; Beynnon et al., 2006; Adachi et al., 2008; Ruedl et al., 2009), potentially due to increased ACL laxity (Chidi-Ogbolu and Baar, 2019). Although, other studies have shown greater ACL injury incidence during the early follicular or late-luteal phase (Myklebust et al., 1998; Slauterbeck et al., 2002). There is often discrepancy in the way that phases are defined and estimated: for example, some studies have only compared injury incidence in pre and post ovulatory phases (Möller-Nielsen and Hammar, 1989; Beynnon et al., 2006; Ruedl et al., 2009, 2011) which does not consider the ~10-fold increase in estrogen concentrations from the early to late follicular phase (Stricker et al., 2006). The interpretation of published injury rates is further hampered by the inclusion of hormonal contraceptive users in data sets (Möller-Nielsen and Hammar, 1989; Myklebust et al., 1998; Wojtys et al., 1998; Slauterbeck et al., 2002; Ruedl et al., 2011; Lefevre et al., 2013) despite the downregulation of endogenous reproductive hormones with hormonal contraceptive use (Elliott-Sale et al., 2013). Further issues with existing evidence include a relatively small number of injuries recorded in studies (<40; Myklebust et al., 1998; Wojtys et al., 1998; Arendt et al., 1999; Slauterbeck et al., 2002; Adachi et al., 2008) and a majority focus solely on ACL injuries. Furthermore, much of this research is in skiers (Beynnon et al., 2006; Ruedl et al., 2009, 2011; Lefevre et al., 2013) which may lack application to other sports.

To date, only Möller-Nielsen and Hammar (1989) have reported the distribution of wider injuries across the menstrual cycle in footballers over the course of a season, although only 45 injuries were reported in non-contraceptive users. Injury incidence was greater during the pre-menstrual and menstrual period compared to the rest of the cycle, although phases were not clearly defined. Interestingly, this effect was only apparent in those with dysmenorrhea (menstrual pain symptoms), which may provide an additional mechanism to those already described for differences in injury rates between menstrual cycle phases. Injury risk may also be modified by menstrual cycle effects on recovery from exercise (Markofski and Braun, 2014) or disturbances to postural control, kinaesthesia, and neuromuscular co-ordination (Posthuma et al., 1987; Fridén et al., 2005, 2006).

The Relative Energy Deficiency in Sport (RED-S) model, which includes menstrual dysfunction as one of its characteristics, has suggested that low energy availability may increase injury risk in athletes (Mountjoy et al., 2018). When dietary energy availability is limited, key physiological processes such as the menstrual cycle are sacrificed to conserve energy, resulting in oligomenorrhea or amenorrhea, and greater injury incidence has been observed in athletes and military recruits with extended cycle durations (Rauh et al., 2010; Knapik et al., 2013). Moss et al. (2020) showed that 23% of elite female footballers had low (≤ 30 kcal·kg·FFM^−1^·day^−1^) energy availability and menstrual dysfunction has been observed in 9.3–19.3% of elite footballers (Sundgot-Borgen and Torstveit, 2007; Prather et al., 2016). To date, the impact of extended menstrual cycle duration has not been explored in relation to injury risk.

The aim of this study is to assess how menstrual cycle phase and extended menstrual cycle length influence the incidence of injuries in English international footballers using data collected over a 4-year period.

## Methods

### Recruitment

Players selected for the England national team Under 15, 16, 17, 18, 19, 20, 23, and Senior level were asked to participate in the study. Informed consent was obtained before data was gathered, and participants were informed that they can withdraw from the study at any time without consequence. Ethical approval was granted by the Nottingham Trent University Non-Invasive Ethical Review Committee (application number: 116V2). In total, the present study includes eight playing squads over 4 years (2012–2016) of data collection. The study and is comprised of 3,947 individual player camp attendances over 160 international camps.

### Data Collection

Only data collected in relation to injuries sustained while representing their country (in either match-play or training camps) were included in the study. Data were collected and analyzed in line with the international consensus statement on the process of conducting epidemiological studies in professional football (Fuller et al., 2006) and the International Olympic Committee Consensus Statement (Bahr et al., 2020).

### Experimental Design and Protocol Description

Data on injury incidence were collected for the entirety of all international camps from 2012 to 2016 using a case-series design. Any injuries that occurred during the international camps were included in the data analysis. Before the study commenced, all medical support staff for each age group were provided with guidance on why the study was taking place, and information on the definitions of all variables recorded as part of the study.

### Injury

An injury was defined as an occurrence which prevented a player from taking part in training or match-play for one or more days following the injury (Fuller et al., 2006). Injuries sustained outside of formal training and match-play were excluded from analysis. Injury information was recorded by each team's medical support staff and a database was created using an electronic medical record system (The Sports Office, Wigan, United Kingdom). Each injury was classified using the Orchard Sports Injury Classification System (Orchard, 2010) by a medical professional within each team.

### Menstrual Cycle Categorization

Self-reported information on hormonal contraceptive use, menstrual cycle length and the number of days since the start of their last menstrual cycle (first day of menstruation) was provided by players to Football Association support staff upon injury occurrence. Exclusion criteria were pre-menarchal athletes, hormonal contraceptive use, missing data for last menstrual period or hormonal contraceptive use, and self-reported irregular menstrual cycles. Self-reported typical menstrual cycle length was used to estimate the day of peak luteinizing hormone (LH) concentration using the regression equation of McIntosh et al. (1980), rounded to the nearest whole day. Where a range was given to describe regular cycle length, the midpoint of that range was taken as the cycle length. Based upon data from Stricker et al. (2006), peak estrogen concentrations were estimated to occur on the day of LH peak and the two preceding days; this was labeled the late follicular phase. The follicular phase was defined as the time between the first day of the last menses and the late follicular phase. The luteal phase was defined as any time point following the late follicular phase.

### Data Analysis

Descriptive statistics were generated to describe frequencies of total injuries, and different types of injuries, by menstrual cycle phase for eumenorrheic players; defined as those with a regular cycle length between 21 and 35 days (Fehring et al., 2006). Frequencies were also compared between injuries recorded during the reported typical cycle length and those that occurred after the cycle would be expected to have finished (“overdue”). Calculating phase length from cycle length results in variation in follicular and luteal phase lengths across individuals. Therefore, person-days were adopted: the number of days estimated for each phase were summed across injuries, to give a sum for each menstrual cycle phase. These were then used to estimate injury incidence rate per 1,000 person-days for each phase. Injury incident rate ratios were calculated by comparing injury incident rates between phases. Injury incident rates and injury incident rate ratios were not calculated for brain/spinal cord/peripheral nervous system (PNS) or bone injuries due to the limited number of these injuries. Index, subsequent and recurrent injuries were combined for the analysis. Due to the nature of the study design and descriptive aims of the study, null hypothesis testing was not deemed appropriate. Analyses were carried out in Excel (Microsoft Office 365, USA) and RStudio 1.0.153 (R Core Team, 2020).

## Results

Players were excluded due to absence of menarche (*n* = 2), hormonal contraceptive use (*n* = 19), self-reported irregular menstrual cycles (*n* = 12), or missing data for key variables (days since last reported menses [*n* = 21]; hormonal contraceptive use [*n* = 14]). There were 156 eligible injuries from 113 players for inclusion in analyses. Twenty-seven players recorded multiple injuries, ranging from 1 to 7 injuries per player. Age at time of injury ranged from 13 to 35 years (median 17 years).

Self-reported regular cycle length ranged from 18 to 64 days (median 28 days). One participant reported a cycle shorter than 21 days; 13 reported long cycles (>35 days). Excluding these participants, the median cycle length remained 28 days (*n* = 142).

For injuries from eumenorrheic players (regular cycle length between 21 and 35 days) estimated follicular phase ranged in length from 8 to 16 days (median 11 days; *n* = 142), and estimated luteal phase ranged from 13 to 16 days (median 14 days). Late follicular phase was 3 days' duration by definition. There were 41 injuries in the follicular phase, 16 injuries in the late follicular, and 57 injuries in the luteal phase.

Twenty eight of 142 (20%) injuries were reported as occurring after the regular cycle was expected to have resumed (“overdue”). Number of days over normal cycle length at time of injury ranged from 0 to 40 days (median 5 days). Thirty-six percent (10 out of 28) of “overdue” injuries were joint/ligament, compared to 21% (24 out of 114) of injuries in the normal menstrual cycle. Of the 114 injuries that were within the regular eumenorrheic cycle length, 65 (57%) were subsequent or recurrent injuries. Of the 28 injuries that were “overdue,” 12 (43%) were subsequent or recurrent injuries.

Excluding those injuries which occurred in the “overdue” period, the total person-days was 3,241, of which 1,287 (39.7%) were in the follicular phase, 342 (10.6%) in the late follicular, and 1,612 (49.7%) in the luteal phase. Injury incidence rates per 1,000 person-days were 31.9 in the follicular phase, 46.8 in the late follicular and 35.4 in the luteal (Table 1). The injury incidence rate ratio showed the rate of injuries was 47% greater in the late follicular phase compared to the follicular phase and 24% lower in the luteal phase compared to the late follicular phase. Injury incident rate ratios showed that muscle and tendon injury rates were 88% greater in the late follicular phase compared to the follicular phase, with muscle rupture/tear/strain/cramps and tendon injuries/ruptures occurring over twice as often during the late follicular phase compared to other phases. Injury incidence rates for joint and ligament injuries in the luteal phase were approximately double that of the follicular phase and almost treble that of the late follicular phase, although only one injury was recorded for the late follicular phase.

**Table 1:** Number of injuries, injury incidence rates, and injury incidence rate ratios for all injuries and injuries separated by type and sub-type (italics) for eumenorrheic participants.

	**Total number of injures**			**Injury Incidence Rate (per 1000 person-days)**			**Injury Incidence Rate Ratios**		
	**Follicular**	**Late Follicular**	**Luteal**	**Follicular**	**Late Follicular**	**Luteal**	**Late follicular: Follicular**	**Luteal: Follicular**	**Luteal: Late Follicular**
All Injuries	41	16	57	31.9	46.8	35.4	1.47	1.11	0.76
Muscle and tendon	28	14	35	21.8	40.9	21.7	1.88	1.00	0.53
*Muscle rupture/tear/* *strain/cramp*	14	8	17	10.9	23.4	10.5	2.15	0.96	0.45
*Tendon injuries/ruptures*	4	3	7	3.1	8.8	4.3	2.84	1.39	0.49
Joint and Ligament	7	1	16	5.4	2.9	9.9	0.54	1.83	3.41
Brain/Spinal cord/PNS	3	1	2						
Bone	3	0	4						

*PNS, Peripheral nervous system*.

## Discussion

The aim of this study was to assess how menstrual cycle phase and extended menstrual cycle length influence the incidence of injuries. Injury incidence rates were 47 and 32% greater in the late follicular phase compared to the follicular phase and luteal phase, with muscle and tendon injury incidence rates in the late follicular phase being almost double that of the other phases. Furthermore, a relatively large proportion of all injuries (20%) occurred after the expected date of menstruation.

### Menstrual Cycle Phase

The potentially greater injury rate in the late follicular phase is consistent with some studies showing a greater incidence of ACL injuries during this phase (Adachi et al., 2008; Ruedl et al., 2009), although we did not limit injury observations to ACL injuries as in previous research. In fact, throughout the observation period, only one ACL rupture occurred during international matches or training in this population, and this in an OC user, so is not included in the analysis. It has been suggested that higher ACL injury rates in the late follicular phase may be a result of reduced ligament stiffness which compromises joint stability (Shultz et al., 2005; Myer et al., 2008; Chidi-Ogbolu and Baar, 2019). In contrast, tendon stiffness may increase injury risk as eccentric load is increased in the muscle with less compliant tendons (Chidi-Ogbolu and Baar, 2019). We showed that, compared to the follicular and luteal phases, muscle and tendon injuries were approximately twice as common during the late follicular phase, when estrogen concentrations are highest (Stricker et al., 2006). This is despite some studies showing that estrogen is negatively associated with tendon stiffness (Bell et al., 2012) and tendon stiffness is lowest in the late follicular phase (Eiling et al., 2007; Casey et al., 2014), although other studies show no change in tendon stiffness across the menstrual cycle (Burgess et al., 2009; Kubo et al., 2009). Therefore, it is not possible to relate muscle and tendon injury rate in the late follicular phase to changes in musculotendinous stiffness. This current study is also in contrast to the only prior study to assess menstrual cycle phase effects on all injuries (Möller-Nielsen and Hammar, 1989), which reported an increased risk of injury during the premenstrual and menstrual period. Möller-Nielsen and Hammar (1989) observed a limited number of injuries (*n* = 45) and did not stratify injury by type or clearly define phases, so direct comparisons are difficult. In the current study, injury incidence rates were lowest in the follicular (31.9 per 1,000 person days) and luteal (35.4 per 1,000 person days) phases, which does not suggest an increase in injury risk pre-menstruation or during menstruation.

### Extended Menstrual Cycles

The proportion of injury in athletes when “overdue” (20%) is relatively high compared to the 9.3–19.3% prevalence of menstrual dysfunction in footballers (Sundgot-Borgen and Torstveit, 2007; Prather et al., 2016), given that time “overdue” will account for a small proportion of these athletes' overall training/match exposure time. However, as menstrual cycle details were only collected upon the occurrence of an injury, it is not possible to identify whether there is an increased risk of injury when menstrual cycle length was extended as there is no “exposure” data for comparative assessment. The range (0–40 days) and median (5 days) time “overdue” at injury, suggest clustering of injury occurrence in the initial days following the expected date of menstruation, potentially highlighting this as a timeframe where injury incidence is relatively high. Previous research has shown that injuries are more common in those with oligomenorrhea and amenorrhea (Rauh et al., 2010; Knapik et al., 2013); however this is the first study to assess injuries in “overdue” athletes in a prospective assessment by cycle date. It is also worth noting that the type of injury was seemingly different when cycle length was extended as 36% (10 out of 28) of “overdue” injuries were joint/ligament, compared to 21% (24 out of 114) of injuries occurring during the typical time frame of the athletes' menstrual cycles This evidence adds to existing data showing the potential negative impacts of menstrual dysfunction on athlete health and performance (Mountjoy et al., 2018).

### Limitations

There are several limitations to this study which should be noted when interpreting these data. Whilst the reported number of injuries in this analysis is substantially greater than previously published research in team sports, numbers are still relatively small, especially when breaking incidence down by injury type which may be differentially affected by the menstrual cycle. However, collecting data of this type on a greater number of international footballers would be impractical without multicentre research teams. The data also include observations which are not independent (e.g., recurrent injuries) and therefore should be interpreted with caution. Self-reported menstrual cycle length was used to predict the time within the menstrual cycle that the injury occurred which can be inaccurate (Jukic et al., 2007) and may vary over time or seasonally with the demands of training (Sundgot-Borgen and Torstveit, 2007). Data quality would be greatly enhanced by players using menstrual cycle tracking applications. Ideally, blood samples would be collected upon injury to confirm hormone concentrations and menstrual cycle phase, although this is less feasible in the type of large-scale project required to assess injuries in team sports. A regression equation was used (McIntosh et al., 1980) to allocate a menstrual cycle phase given the relatively fixed luteal phase duration, although there is some interindividual variation in cycle length (Fehring et al., 2006). By counting days to determine phases, sub-clinical menstrual dysfunctions such as luteal phase defects cannot be identified which could affect interpretation (Schaumberg et al., 2017). We excluded athletes that reported typically irregular menstrual cycles (i.e., oligomenorrhea, amenorrhea, polymenorrhea) as we had no way to estimate cycle phase or whether they were “overdue,” but this should be explored in future research. A more accurate assessment of risk could also be determined by knowing the exposure to training/matches within each menstrual cycle phase as an assumption of the data is that this exposure was even although this could not be determined. Despite these cautionary notes, these data provide an important insight into the pattern of injuries sustained at an elite level of women's football, spanning across a large age range of international competitors.

### Practical Application

This study shows that the incidence of injuries may vary across the eumenorrheic menstrual cycle and that the impact of the menstrual cycle may be dependent upon type of injury or the tissue affected. As this research is in its infancy, we do not recommend that this data is used to inform exercise practice or participation as further work is needed before clear guidelines on the menstrual cycle phase and injury risk mitigation can be generated. This study can, however, serve as basis for future research to develop knowledge in this important area. We showed that a relatively large proportion (20%) of all injuries occurred after the expected date of menstruation. In line with other recommendations (Martin et al., 2018; Armour et al., 2020), we suggest that athletes/practitioners should monitor menstrual cycle length using tracking systems/applications as an “overdue” cycle is easily identified and may present with increased risk for the athlete. Identification of extended cycles can facilitate discussions with appropriate support staff (e.g., medical staff, nutritionist, psychologist) to promote the health and well-being of the athlete.

### Conclusion

This is the first study to assess the injury rate of all injuries by menstrual cycle phase whilst stratifying by injury type and the first study to report the data on the injury rate when “overdue.” These data suggest that muscle and tendon injuries may occur approximately twice as often in the days preceding ovulation. Furthermore, this study has provided initial evidence that injury risk may be elevated in typically eumenorrheic women in the days after their next menstrual cycle was expected to start. This research provides further evidence of the need to consider the menstrual cycle and menstrual dysfunction in athletic populations.

## Data Availability Statement

The raw data supporting the conclusions of this article will be made available by the authors, without undue reservation.

## Ethics Statement

The studies involving human participants were reviewed and approved by Nottingham Trent University Ethics Committee. Written informed consent to participate in this study was provided by the participants' legal guardian/next of kin.

## Author Contributions

CC, JA, RM, and AT conceptualized and designed the study and acquired the data. KT and DM conducted data analysis. DM and IV drafted the manuscript. DM, KT, IV, and CC revised the manuscript. All authors contributed to the content and approved the final version.

## Conflict of Interest

JA, RM, AT, and CC are employees of the English Football Association. IV has received funding as principle investigator from the English Football Association as an Injury Surveillance consultant. The remaining authors declare that the research was conducted in the absence of any commercial or financial relationships that could be construed as a potential conflict of interest.
