# Corrigendum: Injury Incidence Across the Menstrual Cycle in International Footballers

**DOI:** 10.3389/fspor.2021.745792

**Published:** 2021-08-18

**Authors:** Dan Martin, Kate Timmins, Charlotte Cowie, Jon Alty, Ritan Mehta, Alicia Tang, Ian Varley

**Affiliations:** ^1^School of Sport and Exercise Science, University of Lincoln, Lincoln, United Kingdom; ^2^The Football Association, London, United Kingdom; ^3^Department of Sport and Exercise Science, Nottingham Trent University, Nottingham, United Kingdom

**Keywords:** epidemiology, menstrual cycle, injury, soccer, football, female athlete

**Incorrect Acknowledgment**

In the published article, there was an error in the acknowledgements section (page 5, final paragraph). Instead of “**We thank Dr. Colin Fuller (Colin Fuller consultancy) for his work in originally designing and running the Injury and Illness Surveillance program at the FA and Dr. Aileen Taylor for their contribution to the auditing of injury and illness within the Football Association and their assistance with the data processing**,” there should be no acknowledgement given.

The authors apologize for this error and state that this does not change the scientific conclusions of the article in any way. The original article has been updated.

## Publisher's Note

All claims expressed in this article are solely those of the authors and do not necessarily represent those of their affiliated organizations, or those of the publisher, the editors and the reviewers. Any product that may be evaluated in this article, or claim that may be made by its manufacturer, is not guaranteed or endorsed by the publisher.

